# A comparative study of baked and traditional fermented milk: physicochemical characteristics and biological effects in wistar albino rats

**DOI:** 10.1038/s41598-026-60278-3

**Published:** 2026-07-07

**Authors:** Rehab S. Sayed, Sameh S. El-Hadad, Eman S. Ibrahem, Laila K. Hassan, Mahmoud Abd El-Aziz

**Affiliations:** 1 Regional Center for Food & Feed (RCFF), Agricultural Research Center, Giza, Egypt; 2 Dairy Department, Food Industries & Nutrition Research Institute, National Research Centre, Giza, Egypt

**Keywords:** Baked fermented milk, Physicochemical characteristics, Probiotic bacteria, Biological parameters, Biochemistry, Biotechnology, Microbiology, Physiology, Zoology

## Abstract

Baked fermented milk (BFM) is produced by heating milk at high temperatures before fermentation, which promotes Maillard reactions. These reactions enhance sensory and functional properties and generate bioactive compounds; however, they may also lead to the formation of potentially harmful products. This study aimed to evaluate the physicochemical properties and biological effects of BFM, with and without probiotic supplementation, compared with traditional fermented milk (TFM). Cow’s milk was either heated at 90 °C for 5 min or baked at 115 °C for 20 min before inoculation with starter cultures. Half of each preparation was supplemented with probiotics, yielding four formulations: TFM, probiotic TFM (PTFM), BFM, and probiotic BFM (PBFM). Thirty-five male Wistar albino rats were randomly assigned to five groups and fed experimental diets for 45 days, including a control group receiving liquid milk and four groups receiving fermented milk formulations. Physicochemical analysis showed that BFM had higher 5-HMF content and stronger antioxidant activity than TFM, along with slightly higher diacetyl levels but lower apparent viscosity. Biologically, BFM reduced serum glucose levels and oxidative stress markers without adversely affecting body weight gain (BWG), serum lipid profiles, or protein levels. However, rats fed BFM exhibited the highest serum ASAT activity and urea levels. Probiotic supplementation with *Bifidobacterium bifidum* NRRL B-41,410 and *Lacticaseibacillus rhamnosus* NRRL B-442 provided synergistic benefits in the PBFM group, including further reductions in glucose levels, improved lipid profiles, decreased oxidative stress, lower BWG, and mitigation of elevated serum urea and ASAT activity. Overall, although daily consumption of BFM may provide health benefits, it may also induce mild metabolic alterations, which are effectively alleviated through probiotic supplementation.

## Introduction

Fermented or cultured milk has been part of human diets for thousands of years across many cultures and is produced through the controlled fermentation of milk by lactic acid bacteria (LAB) and, in some cases, yeasts. This fermentation process not only preserves milk but also improves its nutritional value and sensory properties. It increases the bioavailability of proteins, vitamins, and minerals while lowering lactose levels, thereby improving digestibility for lactose-intolerant individuals^1^. Beyond their nutritional benefits, fermented milks are well known for their functional and health-promoting properties^2^. Because of these health advantages and their appealing sensory characteristics, fermented milk products occupy an important place in human nutrition and the global dairy industry. A diverse range of fermented milks is produced worldwide, with approximately 400 different names used for traditional and industrial varieties, classified according to fermentation processes, processing methods, and the microorganisms’ involved^3^.

Baked fermented milk (BFM), known as “Ryazhenka” in regions of Russia, Belarus, and Ukraine, is a distinctive dairy product produced by subjecting milk to prolonged high-temperature treatment (95–100 °C for 3–5 h or 115 °C for 15–20 min). This process induces the Maillard reaction between milk sugars and proteins, giving the milk its characteristic brown color, caramel-like aroma, and distinct flavor profile even before fermentation^4^. Following this baking process, the milk is cooled and inoculated with LAB starter cultures for fermentation, yielding a product with a unique taste, aroma, sensory profile, and metabolomic characteristics. The Maillard reaction contributes not only to the development of color and flavor but also to the formation of bioactive compounds that can significantly influence the nutritional and functional properties of foods during processing and storage^5^. Oh et al.^6^ reported that fermented Maillard reaction products (MRPs) may reduce oxidative stress, inhibit platelet aggregation, and decrease both cholesterol synthesis and serum cholesterol levels. These findings highlight their potential role in the prevention of cardiovascular diseases and suggest promising applications in the pharmaceutical and dairy industries. In addition, probiotic microorganisms have been associated with improved digestion, immune modulation, vitamin synthesis, cholesterol reduction, and protection against pathogens^7,8^. Among these microorganisms, *B. bifidum* plays an important role in the human gastrointestinal tract by supporting nutrient metabolism, intestinal barrier integrity, and immune regulation^9^. Similarly, *L. rhamnosus* is known for promoting gut health, enhancing immune function, and managing diarrhea due to its ability to survive gastric acidity, adhere to intestinal cells, and inhibit pathogens^10^. Kim et al.^11^ reported that MRPs derived from milk proteins fermented with *L. rhamnosus* or *L. gasseri* exhibited antimicrobial activity against *C. perfringens* through iron chelation and disruption of bacterial cell membranes. Conversely, several studies have reported that high-heat treatment may also lead to the formation of potentially harmful MRPs^12,13^. Common undesirable MRPs include 5-hydroxymethyl-2-furfural (5-HMF), glyoxal, methylglyoxal, Nε-carboxymethyllysine (CML), and Nε-carboxyethyllysine^12^. Among these compounds, 5-HMF has been associated with cytotoxic, indirectly mutagenic, carcinogenic, hepatotoxic, and nephrotoxic effects^14^. Furthermore, Li et al.^12^ demonstrated that MRPs formed in BFM during heat treatment and fermentation may exert adverse health effects through the generation of advanced glycation end products (AGEs), such as CML. These AGEs are known to promote oxidative stress and inflammation and have been linked to metabolic disorders, including diabetes, as well as vascular and neurodegenerative diseases. Additionally, the formation of these MRPs may reduce the nutritional quality of fermented milk by decreasing the bioavailability of essential amino acids, particularly lysine^12^. This study aimed to evaluate the physicochemical characteristics, biological impacts, and potential toxicological risks associated with MRPs generated during the high-temperature production of BFM at 115 °C for 20 min in comparison with traditional fermented milk (TFM). Unlike previous studies that examined individual MRPs separately, this research investigated their cumulative and synergistic effects within a complex dairy matrix to provide a more realistic and comprehensive assessment of their influence on product quality and consumer safety. In addition, the study investigated whether probiotic supplementation using *B. bifidum* and *L. rhamnosus* could enhance the nutritional and functional properties of BFM, improve its biological activity, and mitigate the formation or effects of harmful thermal byproducts.

## Materials and methods

### Materials

Fresh cow’s milk was obtained from the Faculty of Agriculture farm (Cairo University, Egypt). Probiotic strains (*Bifidobacterium bifidum* NRRL B-41410 and *Lacticaseibacillus rhamnosus* NRRL B-442) and starter cultures (*Lactobacillus delbrueckii* subsp. *bulgaricus* and *Streptococcus salivarius* subsp. *thermophilus*) were supplied from the stock cultures of the Dairy Microbiology Laboratory (National Research Centre, Giza, Egypt). Zinc sulfate, potassium hexacyanoferrate, 5-hydroxymethyl-2-furfural (5-HMF), semicarbazide hydrochloride, 2,2-diphenyl-1-picrylhydrazyl (DPPH), and 2,2’-azino-bis(3-ethylbenzothiazoline-6-sulfonic acid) (ABTS) were purchased from Sigma-Aldrich (St. Louis, MO, USA). HPLC-grade methanol was obtained from Merck (Darmstadt, Germany). Standard rodent pellets, containing 20.0% protein, 0.3% DL-methionine, 5.0% maize oil, 5.1% fiber, 4.7% mineral mix, and 1.0% vitamin mix, were purchased from Miladco Company (El-Obour City, Cairo, Egypt). All chemicals and reagents used were of analytical grade. Thirty-five male Wistar albino rats (*Rattus norvegicus*), weighing 119 ± 10.5 g, were obtained from the Animal House Colony (National Research Centre, Giza, Egypt).

### Methods

#### Making of traditional and baked fermented milk

Fresh cow’s milk (3.3% fat, 3.19% protein, 4.87% lactose, and 0.71% ash) was preheated to 65 °C and homogenized with a laboratory homogenizer (Polytron^®^ PT 10–35 GT, Kinematica, Switzerland) at 21,000 rpm for 5 min. The homogenized milk was divided into two equal portions. One portion was heated in a water bath at 90 °C for 5 min (heat-treated), while the other was autoclaved at 115 °C for 20 min. Both portions were then cooled to 42 °C using cold water before being inoculated with 2% (w/w) mixed starter cultures (*Lb. bulgaricus* and *S. thermophilus*, 1:1). Each portion was further divided in half, and one half of each was supplemented with 2% (w/w) mixed probiotic cultures (*B. bifidum* and *L. rhamnosus*, 1:1), yielding in four formulations: traditional fermented milk (TFM), probiotic traditional fermented milk (PTFM), baked fermented milk (BFM), and probiotic baked fermented milk (PBFM). Fermentation proceeded at 42 °C until the pH reached 4.6–4.7, after which the curd was gently stirred to form a uniform product. The fermented milk was then dispensed into sterile glass bottles, each containing 150 ml. Samples were collected for analysis on days 1, 7, and 14 during storage at 5 ± 1 °C. All treatments were prepared in three independent batches.

#### Physicochemical analysis

##### Measurement of pH

The pH of the stirred fermented milk (FM) samples during storage was measured using a laboratory pH meter equipped with a glass electrode (HANNA, Portugal). Before the measurements, the electrode was calibrated with standard buffer solutions at pH 4.01, 7.00, and 10.01.

##### Determination of flavor compounds

Acetaldehyde and diacetyl, key flavor compounds, were determined in the stirred FM samples using the Conway microdiffusion–semicarbazide method, as described by Lees & Jago^15^. Briefly, 1 ml of semicarbazide solution (6.7 mM) was added to the inner well of a Conway cell to serve as a trapping agent for carbonyl compounds. A 3 g chilled FM sample was placed in the outer well, immediately sealed with a glass lid, and incubated at 30 °C for 90 min. The absorption liquid was then transferred to a 10 ml volumetric flask and diluted to volume with distilled water. The resulting semicarbazones were measured using a Shimadzu UV-Vis 1201 spectrophotometer at 224 nm for acetaldehyde and 270 nm for diacetyl. Concentrations were calculated using standard calibration curves (5–100 µmol/100 g), and all analyses were performed in triplicate.

##### Determination of 5-hydroxymethyl-2-furfural (5-HMF)

5-HMF was extracted from the stirred FM following the method of El-Hadad et al.^16^. Briefly, in a 15 ml centrifuge tube, 5 ml of ethanol was added to 2 g of FM sample. The mixture was vortexed for 2 min and then centrifuged at 6000 ×g for 20 min. To the supernatant, 250 µL of Carrez I reagent (15% potassium hexacyanoferrate) and 250 µL of Carrez II reagent (30% zinc sulfate) were added. The mixture was then transferred to another centrifuge tube and centrifuged again at 6000 ×g for 20 min. Finally, 2 ml of the clear supernatant were collected, dried at 40 °C under nitrogen using a blowing concentrator, and re-dissolved in 2 ml of distilled water. UPLC H-Class Waters (Detector PDA, Column C_18_ Phenomenex; 150 mm × 4.6 mm × 5 μm) were used to measure the 5-HMF in purified samples. A mobile phase consisting of a 1:4 v/v mixture of acetonitrile and water was used for 20 min at a flow rate of 0.6 ml/min. The injection volume was 20 µL, and the UV detection was performed at a wavelength of 284 nm. The concentration of 5-HMF was determined by comparing the UV spectra and retention times of the samples with those of standard solutions. Peak areas from standard concentrations ranging from 2.5 to 25 µg/ml were used to construct a calibration curve.

##### Antioxidant activity

The antioxidant activity of the stirred FM samples was assessed in filtered whey using stable DPPH and ABTS radical scavenging assays, based on the methods of Brand-Williams et al.^17^ and Re et al.^18^, respectively. For each assay, 100 µL of whey was mixed with 3.9 ml of the respective working solution: DPPH (25 mg/L in methanol) or ABTS (7 mM ABTS with 2.45 mM K₂S₂O₈). After incubation in the dark at room temperature for 30 min, the degree of decolorization was measured using a UV-Vis spectrophotometer (Shimadzu UV-Vis 1201, Japan) at 517 nm for DPPH and 734 nm for ABTS. Control solutions without whey were prepared for both assays. The scavenging activities of DPPH and ABTS were calculated using the following formula:

$${\text{FM antioxidant activity }}\left( \% \right){\text{ }} = {\text{ }}[({\mathrm{A}}_{0} {-}{\text{ A}}_{{\mathrm{1}}} )/{\mathrm{A}}_{0} ]{\text{ x 1}}00$$

Where: A_0_ represents the absorbance of the control solution, and A_1_ represents the absorbance of the FM sample.

##### Apparent viscosity

The rheological properties of stirred FM samples were evaluated using a Brookfield digital viscometer (Model DV-II, Canada) equipped with an LV-04 spindle, following the methodology described by Shazly et al.^19^. Samples were gently agitated and transferred to a 100 ml glass cylinder for analysis at a controlled temperature of 7 ± 1 °C. To characterize the flow behavior, apparent viscosity was measured at increasing spindle speeds (4 to 50 rpm). For each interval, readings were recorded after 30 s of continuous shearing and expressed in Pascal-seconds (Pa·s). The flow behavior index (*n*) was calculated by fitting the experimental flow data to the Power Law (Ostwald-de Waele) model. All measurements were performed in triplicate to ensure reproducibility.

#### Biological study

##### Diet preparation

Ground standard rodent feed pellets (normal diet; ND) were thoroughly mixed with liquid cow’s milk (LM), TFM, PTFM, BFM, or PBFM at a 2:1 ratio to prepare five experimental diets, designated as NDLM, NDTFM, NDPTFM, NDBFM, and NDPBFM, respectively. Each mixture was shaped into small pellets and dried overnight at room temperature (27 ± 2 °C) under gentle airflow. Following dehydration, the formulated diets shared an identical nutritional profile on a dry matter basis: 22.43% crude protein, 6.92% ether extract, 5.31% crude fiber, 5.26% ash, and 58.72% carbohydrates. The viable counts of *Lb. bulgaricus* and *S. thermophilus* were determined according to the method of Dave & Shah^20^, while *L. rhamnosus* and *B. bifidum* were tested using the method described by Tharmaraj & Shah^21^. The minimum viable count of each bacterial strain in the diets was maintained at least 10⁸ cfu/g of feed.

##### Experimental design

The animals were housed in suitable plastic cages and provided with a standard rodent diet and tap water for one week prior to the start of the study. They were maintained under controlled conditions of 25 ± 2 °C air temperature and a 12 h light–dark cycle. Following the adaptation period, the animals were weighed and randomly allocated to five groups for a 45-day experimental period: Group 1 (control) received NDLM, while Groups 2–5 received NDTFM, NDPTFM, NDBFM, or NDPBFM, respectively. To ensure comparable baseline nutritional composition among all groups, liquid milk was incorporated into the normal diet at proportions equivalent to those used in the experimental diets. This design minimized differences in macronutrient intake, allowing any observed biological or physiological effects to be attributed primarily to bioactive compounds generated through thermal processing, starter culture fermentation, or probiotic activity, rather than to variations in nutrient composition. The rats were fed the experimental diet *ad libitum*; approximately 20 g of formulated diet per rat was provided daily, with free access to water at all times. The study protocol was approved by the Ethical Committee for Animal Care and Use at the Faculty of Science, Al-Azhar University, Egypt (Approval No. AZHAR 10/2025, January 2025).

##### Animal weighing and blood sampling

At the end of the experimental period, all rats were fasted overnight (12 h) and weighed. Blood samples were then collected under anesthesia using isoflurane inhalation. The samples were collected into clean Wasserman tubes and were allowed to clot at room temperature for 30 min. Serum was subsequently separated by centrifugation at 3000 rpm for 5 min, aliquoted, and stored at − 80 °C until further biochemical analyses could be performed.

##### Growth performance and feed efficiency

Rats’ body weights (g) were recorded on day 1 (initial body weight, IBW) and day 45 (final body weight, FBW). Body weight gain (BWG, %) was then calculated as follows:

$${\text{BWG }}\left( \% \right){\text{ }} = {\text{ }}\left[ {\left( {{\text{FBW }}{-}{\text{ IBW}}} \right)/{\mathrm{IBW}}} \right]{\text{ x 1}}00$$

Daily feed intake (FI) was calculated by subtracting the mass of the residual feed from the total feed offered and was expressed as g/rat/day. Feed efficiency (FE) was defined as the ratio of BWG to cumulative FI^22^. It was calculated using the following equation and expressed as grams of weight gain per gram of feed intake (g/g):

$${\text{FER }}\left( {{\mathrm{g}}/{\mathrm{g}}} \right){\text{ }} = {\text{ }}\left( {{\text{FBW }}{-}{\text{ IBW}}} \right)/{\mathrm{FI}}$$

##### Biochemical analyses

Serum glucose, total cholesterol (TC), triglycerides (TGs), low- and high-density lipoproteins (LDL-c and HDL-c), urea, creatinine, total protein (TP), albumin, malondialdehyde (MDA), and nitric oxide (NO) levels as well as alanine aminotransferase (ALAT) and aspartate aminotransferase (ASAT) activities were measured spectrophotometrically according to the instructions provided with the Biodiagnostic reagent kits (Biodiagnostic, Dokki, Giza, Egypt).

#### Statistical analysis

Data were analyzed using SAS version 22 (SAS Institute Inc., Cary, NC, USA) using the General Linear Model (GLM) procedure. Prior to analysis, the normality of residuals was assessed using the Shapiro-Wilk test, and homogeneity of variance was evaluated to verify compliance with ANOVA assumptions. For the physicochemical characteristics of stirred FM during storage, a two-factor completely randomized design was used. The statistical model included treatment (T_i_), storage period (S_j_), and their interaction (T × S)_ij_ as fixed effects: Y_ijk_ = µ + T_i_ + S_j_ + (T × S)_ij_ + e_ijk_ where Y_ijk_ is the observed response, µ is the overall mean, T_i_ is the fixed effect of treatment, S_j_ is the fixed effect of storage period, (T × S)_ij_ is the interaction between treatment and storage period, and e_ijk_ is the residual error. Biological responses in the animal experiment were analyzed using a one-way ANOVA with treatment as the fixed effect according to the following model: Y_ij_ = µ + T_i_ + e_ij_ where Y_ij_ is the observed response, µ is the overall mean, T_i_ is the fixed effect of treatment, and e_ij_ is the residual error. When significant effects were detected, means were separated using Duncan’s multiple range test. Although this procedure is less conservative than some alternative multiple-comparison methods, it is widely used in animal nutrition and food science research because it provides adequate sensitivity for detecting treatment differences in balanced experimental designs with a limited number of treatments. Statistical significance was declared at *P* < 0.05. Data are presented as means with pooled SEM based on three independent experiments for physicochemical characterization and seven rats per treatment group for the biological study.

## Results and discussion

### Physicochemical characteristics

#### Changes in pH and flavor compounds

Table 1 presents selected biochemical parameters, including pH and the flavor compounds acetaldehyde and diacetyl, in baked fermented milk (BFM) and probiotic baked fermented milk (PBFM) during 14 days of storage at 5 ± 1 °C, compared with traditional fermented milk (TFM) and probiotic traditional fermented milk (PTFM). No significant differences in pH were observed between BFM and TFM at any storage time, regardless of probiotic addition. Although probiotic FM samples (PTFM and PBFM) showed slightly lower pH values than their non-probiotic counterparts (TFM and BFM), these differences were not statistically significant. Similar results were reported by Ayyash et al.^23^ for cow’s milk fermented with *Lb. plantarum* or *Lb. salivarius* after various heat or pressure treatments. During 14 days of cold storage, all FM samples exhibited a significant decline in pH (*P <* 0.001), reaching approximately 4.13–4.27. This trend has also been observed in synbiotic low-fat yoghurt and probiotic brown yoghurt^16,24^. The gradual decrease in pH is attributed to continued bacterial metabolism of lactose and the production of organic acids, mainly lactic acid and acetic acid.

Regarding flavor compounds, BFM samples showed lower acetaldehyde concentrations than TFM, with a significant difference observed only on day 1, while diacetyl levels were slightly higher in BFM. These results suggest that intense heat treatment may reduce acetaldehyde formation while promoting diacetyl production. These findings contrast with those reported by Kurultay et al.^25^, who observed different acetaldehyde profiles in yoghurt produced using single and mixed starter cultures. Similarly, Li et al.^26^ found that heating milk can increase both acetaldehyde and diacetyl levels in fermented milk, especially when higher temperatures and longer heating times are applied. The addition of probiotic cultures (*L. rhamnosus* and *B. bifidum*) further reduced acetaldehyde levels and increased diacetyl compared with non-probiotic fermented milk. The differences were significant on day 1 for acetaldehyde and on day 14 for diacetyl. A similar observation was reported by Masiá et al.^27^ in plant-based raw materials fermented with *L. rhamnosus*. This effect may be attributed to the ability of *L. rhamnosus* to metabolize acetaldehyde via aldehyde dehydrogenase, converting it into acetate, as reported by Trachootham et al.^28^. During storage, acetaldehyde levels decreased significantly (*P* = 0.022), whereas diacetyl levels increased (*P* = 0.048), possibly due to the oxidation of acetaldehyde and its subsequent conversion into other compounds such as acetone or ethanol. Similar trends have been reported in previous studies^16,19^.

**Table 1:** pH values and concentrations of flavor compounds (acetaldehyde and diacetyl) in baked and traditional fermented milk samples, with and without probiotic, during 14 days of storage at 5 ± 1 °C.

Items	Storage period (day)	Fermented milk treatments				SEM	*P-*value		
		TFM	PTFM	BFM	PBFM		TRT	STG	TRT x STG
pH	1	4.61^Aa^	4.50^Aa^	4.59^Aa^	4.49^Aa^	0.031	0.421	< 0.001	0.991
	7	4.36^Ab^	4.25^Ab^	4.31^Ab^	4.22^Ab^				
	14	4.27^Ab^	4.19^Ab^	4.19^Ab^	4.13^Ab^				
Acetaldehyde (µmol/100 g)	1	92.71^Aa^	81.59^Ba^	82.67^Ba^	71.75^Ca^	2.701	0.022	< 0.001	0.725
	7	77.42^Ab^	64.50^Bb^	70.79^ABb^	63.50^Bb^				
	14	56.00^Ac^	48.83^Ac^	52.50^Ac^	47.83^Ac^				
Diacetyl (µmol/100 g)	1	27.00^Ac^	29.39^Ac^	29.81^Ac^	31.59^Ac^	2.152	0.048	< 0.001	0.706
	7	36.01^Bb^	43.00^ABb^	41.83^BAb^	45.14^Ab^				
	14	51.79^Ba^	60.28^ABa^	56.54^BCa^	64.35^Aa^				

SEM: standard error of the mean. Means with the same uppercase letters within a row (among treatments) or the same lowercase letters within a column (among storage) are not significantly different (*P* < 0.05). TFM, traditional fermented milk; PTFM, probiotic traditional fermented milk; BFM, baked fermented milk; and PBFM, probiotic baked fermented milk.

#### Changes in 5-HMF

**Fig. 1:**
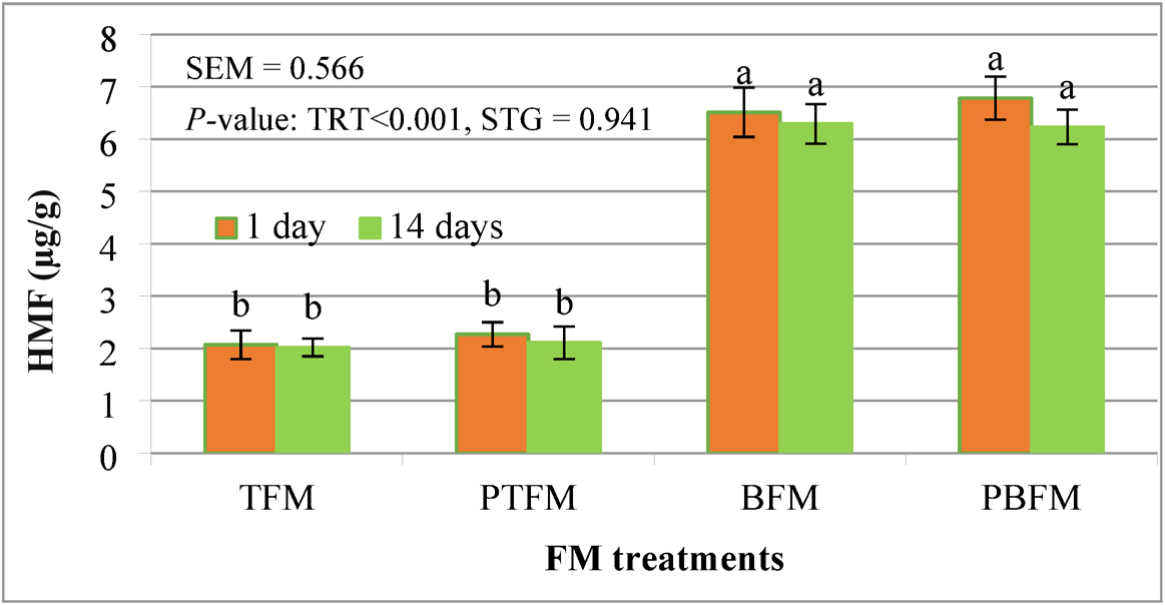
5-HMF concentrations (µg/g) in baked and traditional fermented milk, with and without probiotics, during 14 days of storage at 5 ± 1 °C. TFM, traditional fermented milk; PTFM, probiotic traditional fermented milk; BFM, baked fermented milk; PBFM, probiotic baked fermented milk.

5-HMF was determined as a representative marker of MRPs due to its well-established association with thermal processing and sugar degradation. Because 5-HMF accumulation directly reflects the advanced stages of heat-induced pathways, its concentration serves as a reliable proxy for the formation of other complex MRPs—such as glyoxal, methylglyoxal, furosine, and Nε-carboxymethyllysine,—which is consistent with observations in thermally processed matrices like cookies and milk^29^. As expected, BFM exhibited significantly higher levels of 5-HMF (*P <* 0.001), the compound responsible for its characteristic brown color, compared with TFM (Fig. 1). Specifically, 5-HMF concentrations in both BFM and PBFM ranged from 6.29 to 6.78 µg/g, whereas TFM and PTFM showed considerably lower values (2.02–2.27 µg/g). These findings are consistent with the range reported by El-Hadad et al.^16^ for brown yoghurt produced from buffalo milk heated for 4 h at 97 ± 1 °C. However, the values observed in the present study exceed those reported by Li et al.^4^ for standardized cow’s milk. 5-HMF is a typical heat-induced compound formed under acidic conditions either as an intermediate of the Maillard reaction or via sugar dehydration^30^. However, the incorporation of probiotic strains *B. bifidum* and *L. rhamnosus* did not significantly alter 5-HMF levels during fermentation. Furthermore, refrigerated storage did not result in significant changes in 5-HMF content, although a slight decline was observed across all treatments by day 14 compared with day 1. This observation aligns with previous findings by Shapla et al.^31^, who reported stable 5-HMF levels in UHT milk stored at 4 and 8 °C, in contrast to increased formation at room temperature. The slight reduction in 5-HMF during storage may reflect a balance between its formation and degradation pathways, which are influenced by environmental factors such as temperature and moisture. For instance, 5-HMF can undergo further reactions, including acylation, oxidation, and hydrogenation, leading to the formation of various high-value derivatives^32^.

#### Antioxidant activity

Table 2 shows the DPPH and ABTS radical-scavenging activities of BFM and TFM, with and without probiotic supplementation, during 14 days of storage at 5 ± 1 °C. Overall, BFM exhibited significantly higher antioxidant activity against both DPPH and ABTS radicals compared with TFM (*P <* 0.001). On day 1, BFM showed DPPH and ABTS scavenging activities of 22.12 and 31.42%, respectively, while TFM recorded significantly lower values of 11.76 and 22.19%. The enhanced antioxidant activity observed in heat-treated milk can be attributed to several factors, including the formation of MRPs^33^, protein denaturation leading to the release of antioxidant peptides, and the generation of novel antioxidant compounds such as lactulose^33,34^. The addition of probiotic strains *B. bifidum* and *L. rhamnosus* resulted in a slight, yet statistically non-significant, increase in the antioxidant activities of PBFM and PTFM compared with their respective controls. Similar observations have been reported by Shazly et al.^19^ for probiotic yoghurt and by El-Hadad et al.^16^ for probiotic brown yoghurt. During fermentation, probiotic bacteria hydrolyze milk proteins, releasing peptides with antioxidant properties, as demonstrated for water-soluble peptide fractions isolated from probiotic yoghurt^35^. Furthermore, the proteolytic activity of LAB and probiotic cultures may persist during storage, contributing to the continuous generation of bioactive peptides and a gradual enhancement of antioxidant capacity over periods of up to 20 days^36^.

**Table 2:** Antioxidant activity of baked and traditional fermented milk, with and without probiotics, during 14 days of storage at 5 ± 1 °C.

Items	Storage period (day)	Fermented milk treatments				SEM	*P*-value		
		TFM	PTFM	BFM	PBFM		TRT	STG	TRT x STG
DPPH scavenging activity (%)	1	11.76^Ba^	12.10^Ba^	22.12^Aa^	21.91^Aa^	1.224	<0.001	0.433	0.167
	7	12.16^Ca^	15.00^Ba^	21.63^Aa^	21.52^Aa^				
	14	12.86^Ca^	16.27^Ba^	22.21^Aa^	24.13^Aa^				
ABTS scavenging activity (%)	1	22.19^Ba^	25.77^Ba^	31.42^Aa^	34.83^Aa^	1.412	< 0.001	0.331	0.726
	7	22.88^Ba^	27.41^ABa^	29.82^Aa^	35.14^Aa^				
	14	26.91^Ba^	28.41^Ba^	33.99^Aa^	36.18^Aa^				

SEM: standard error of the mean. Means with the same uppercase letters within a row (among treatments) or the same lowercase letters within a column (among storage) are not significantly different (*P* < 0.05).

See footnotes at the bottom of Table 1.

#### Apparent viscosity

**Fig. 2:**
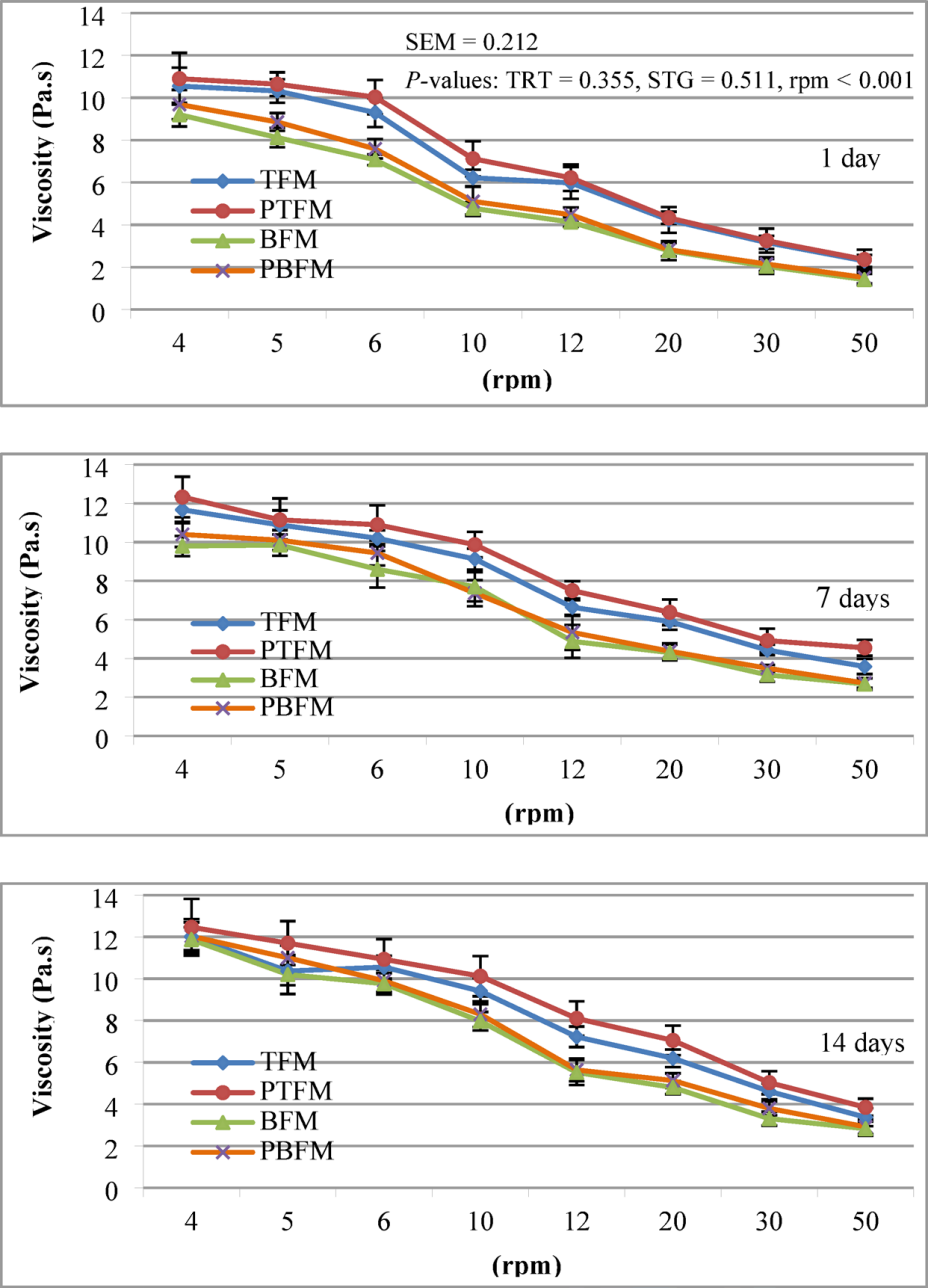
Apparent viscosity of baked and traditional fermented milk, with and without probiotics, during 14 days of storage at 5 ± 1 °C. TFM, traditional fermented milk; PTFM, probiotic traditional fermented milk; BFM, baked fermented milk; PBFM, probiotic baked fermented milk.

Studying the viscosity of FM products is essential for understanding their texture, mouthfeel, gel structure, water-holding capacity, and overall quality. Several studies, including those by Tamime & Robinson^37^, have shown that heating milk at 85–90 °C for 5–30 min or applying UHT treatment can enhance the viscosity of FM. In contrast, excessively severe heat treatments, such as heating at 115 °C for 20 min, may negatively affect viscosity. As illustrated in Fig. 2, all FM samples exhibited non-Newtonian, shear-thinning (pseudoplastic) behavior, characterized by a gradual decrease in apparent viscosity with increasing rotational speed (*P <* 0.001). This behavior was confirmed by the flow behavior index (*n*), which remained consistently below unity (*n* < 1) across all samples throughout storage, ranging from 0.25 to 0.54. On day 1, the *n* values for TFM, PTFM, BFM, and PBFM were 0.37, 0.36, 0.25, and 0.24, respectively, indicating highly pseudoplastic behavior; however, these values increased to 0.51, 0.54, 0.41, and 0.43, respectively, by day 14. The increase in *n* values suggests a reduction in the degree of shear-thinning behavior over time, although all samples retained their non-Newtonian characteristics because *n* remained well below unity. Notably, BFM and PBFM exhibited lower *n* values than TFM and PTFM at all storage times, indicating a stronger pseudoplastic character. Additionally, BFM displayed a lower apparent viscosity than TFM, though this difference was not statistically significant (*P =* 0.335). Excessive or prolonged thermal processing can cause intensive whey protein denaturation and aggregation that hinders proper network formation during fermentation, weakening the acid gel structure compared to heating at approximately 95 °C^38^. Similarly, Ichimura et al.^39^ found that systems treated at 130 °C under 5 MPa form a thinner, less dense protein network, whereas those heated at 95 °C develop a thicker, more interconnected structure. The incorporation of probiotics, including *B. bifidum* and *L. rhamnosus*, resulted in a slight, non-significant increase in apparent viscosity in both FM types, aligning with observations by El-Hadad et al.^16^ in brown yoghurt. The production of capsular polysaccharides by *L. rhamnosus* during fermentation has been confirmed using optical and transmission electron microscopy^32^. Salazar et al.^40^ also showed that *L. rhamnosus* improves the quality of goat milk yoghurt by enhancing microbial growth, acidification, and viscosity development, thereby improving product texture. During storage, the apparent viscosity of all FM samples increased progressively, though the effect of time was not statistically significant (*P =* 0.511). Ongoing metabolic activity and slow post-acidification by LAB at low temperatures induce a gradual decline in pH, strengthening the protein network^41^. The simultaneous increase in both apparent viscosity and *n* values suggests continued structural rearrangement within the acid gel matrix, resulting in a denser and more thermodynamically stable protein network while maintaining pseudoplastic flow behavior.

### Biological parameters

#### Growth performance and feed efficiency ratio

The body weight gain percentage (BWG, %), feed intake (FI, g/rat/d), and feed efficiency ratio (FER, g/g) of rats fed the experimental diets are presented in Table 3. Rats fed NDBFM showed a BWG%, FI, and FER comparable to those fed NDTFM and NDLM. This indicates that intensive heat treatment of cow’s milk (115 °C for 20 min) did not significantly affect these growth parameters compared with moderate heat treatment (90 °C for 5 min). This observation aligns with the findings of Shu et al.^42^, who reported that while different heat treatments of yak milk (65, 90, and 120 °C) altered gut microbiota composition and metabolic profiles, they did not significantly influence overall BWG. Similarly, despite the elevated concentrations of 5-HMF detected in BFM (Fig. 1), long-term administration of 5-HMF (40 or 80 mg/kg for 11 months) has been shown not to affect the final body weight of rats^43^. In contrast, probiotic supplementation with *B. bifidum* and *L. rhamnosus* significantly reduced BWG% in both the NDPTFM and NDPBFM groups compared with their corresponding non-probiotic groups (*P =* 0.010). A similar, although less pronounced, numerical reduction in FI and FER was observed in rats fed NDPTFM and NDPBFM; however, these differences did not reach statistical significance. Because no significant differences were detected in FI (*P =* 0.156) or FER (*P =* 0.245), the reduction in BWG% cannot be explained solely by decreased feed intake or altered feed utilization efficiency. Several mechanisms have been proposed to explain the effects of probiotics on body weight regulation, including modulation of the gut microbiota, reduced lipid absorption, increased fecal fat excretion, attenuation of low-grade inflammation, and stimulation of satiety-related hormones such as peptide YY and glucagon-like peptide-1^44,45^. In addition, probiotic-fermented milk has been reported to enhance calcium bioavailability, which may promote lipolysis and reduce adiposity through regulation of intracellular Ca²⁺ concentrations^46,47^.

**Table 3:** Body weight gain, feed intake, and feed efficiency ratio of rats fed normal diets mixed with baked or traditional fermented milk, with or without probiotic bacteria, over 45 days.

Type of diet	Body weight gain (%)	Feed intake (g/rat/d)	Feed efficiency ratio (g/g)
NDLM	98.92^a^	17.44^a^	0.170^a^
NDTFM	96.15^a^	17.27^a^	0.164^a^
NDPTFM	88.31^b^	16.75^a^	0.160^a^
NDBFM	97.92^a^	17.15^a^	0.168^a^
NDPBFM	89.92^b^	16.59^a^	0.161^a^
SEM	1.124	0.122	0.018
*P-*value	0.010	0.156	0.245

SEM: standard error of the mean. Means with the same lowercase letters within a column are not significantly different (*P* < 0.05). NDLM, normal diet mixed with liquid milk; NDTFM, normal diet mixed with traditional fermented milk; NDPTFM, normal diet mixed with probiotic traditional fermented milk; NDBFM, normal diet mixed with baked fermented milk; NDPBFM, normal diet mixed with probiotic baked fermented milk.

#### Serum glucose

**Fig. 3:**
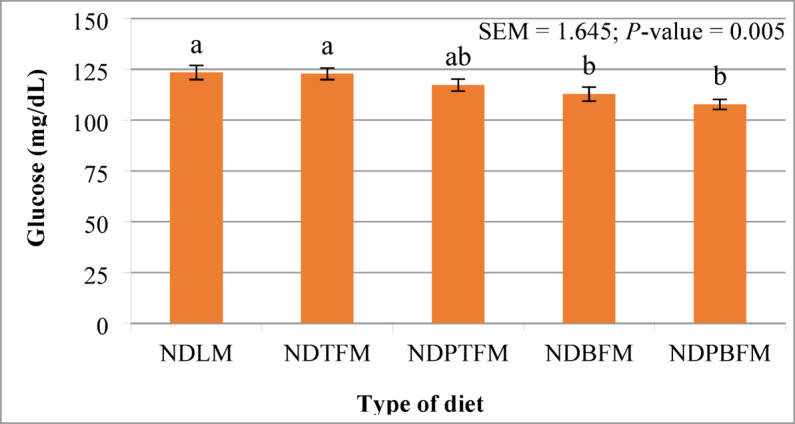
Serum glucose of rats fed normal diets mixed with baked or traditional fermented milk, with or without probiotic bacteria, over 45 days.

Serum glucose levels did not differ significantly among rats fed NDTFM, NDPTFM, and NDLM, although the NDPTFM group showed slightly lower levels than the NDLM and NDTFM groups (Fig. 3). However, rats fed NDBFM or NDPBFM exhibited significantly lower serum glucose levels than those fed NDLM (*P* = 0.005); with the NDPBFM group showing the greatest reduction (12.6%). These effects may be mediated through several probiotic-related mechanisms. *B. bifidum* is a beneficial bacterium capable of producing organic acids, particularly acetate and lactate, which can subsequently be converted by other gut microbiota into butyrate. Butyrate has been shown to inhibit NF-κB activation, thereby reducing the production of pro-inflammatory cytokines such as TNF-α, IL-6, and IL-1β. This anti-inflammatory effect may improve insulin sensitivity in muscle and liver tissues, enhance glucose uptake, and protect pancreatic β-cells from inflammatory damage, ultimately supporting insulin secretion^48^. Additionally, *L. rhamnosus* can downregulate hepatic gluconeogenesis-related genes, leading to reduced endogenous glucose production by the liver^49^. Furthermore, bioactive peptides generated during the fermentation of dairy proteins by LAB (including *L. rhamnosus* and *S. thermophilus*) have demonstrated inhibitory activity against α-amylase, α-glucosidase, and dipeptidyl peptidase IV—key enzymes involved in carbohydrate digestion and glucose metabolism. The inhibition of these enzymes may contribute to lower postprandial blood glucose levels^50^. Intensive heat treatment denatures native milk proteins, exposing additional cleavage sites to bacterial enzymes and consequently promoting the release of greater amounts of bioactive peptides. High-molecular-weight Maillard reaction compounds, particularly melanoidins, may further contribute to reduced intestinal glucose absorption^51^.

#### Serum lipid profile

As shown in Table 4, rats fed normal diets containing FM exhibited lower serum triglycerides (TGs) and total cholesterol (TC) levels than those fed NDLM, although the reduction in TC in the NDBFM group was not statistically significant. The NDPTFM group showed the greatest decreases in TGs and TC; 31.52 and 40.65%, respectively, relative to the NDLM group (*P <* 0.001). Rats fed NDTFM and NDPTFM exhibited the lowest LDL-c levels (*P =* 0.007), while NDTFM resulted in the highest HDL-c levels (*P* = 0.011). Overall, NDTFM was more effective than NDBFM in improving lipid profiles, and this effect was further enhanced by supplementation with *B. bifidum* and *L. rhamnosus*. These findings are consistent with previous studies indicating that fermented milk improves serum lipid profiles, particularly when probiotics are included^52^. The lipid-lowering effects of probiotics may be attributed to several mechanisms. These include increased bile acid excretion, leading to reduced cholesterol absorption, and the activity of bile salt hydrolases produced by certain bacterial genera, which deconjugate bile salts and reduce their enterohepatic recirculation. Cholesterol may also be incorporated into microbial cell membranes during bacterial growth^53,54^. In the present study, NDBFM alone had no marked effect on lipid profiles relative to NDLM, whereas probiotic supplementation improved the lipid-lowering efficacy, particularly for TG and TC levels. Although UHT processing may promote lipid oxidation, hydrolysis, and the formation of free fatty acids compared with milder treatments such as pasteurization, clinical studies in adults indicate no significant differences in blood TC, LDL-c, HDL-c, or TGs levels between UHT-treated and pasteurized milk^55^. Additionally, the fatty acid profile and bioactive constituents of Ryazhenka (BFM) are comparable to those of kefir, a product well known for its lipid-regulating properties. This suggests that the type of heat treatment has a minimal impact on postprandial lipid responses in healthy individuals.

**Table 4:** Serum lipid profile of rats fed normal diets mixed with baked or traditional fermented milk, with or without probiotic bacteria, over 45 days.

Type of diet	Serum lipid profile			
	Triglycerides (mg/dl)	Cholesterol (mg/dl)	LDL-c (mg/dl)	HDL-c (mg/dl)
NDLM	172.71^a^	70.58^a^	73.05^a^	74.27^ab^
NDTFM	146.07^c^	63.52^b^	62.97^b^	82.08^a^
NDPTFM	131.31^d^	50.18^c^	63.29^b^	71.23^b^
NDBFM	168.07^ab^	70.84^a^	70.53^a^	67.32^b^
NDPBFM	161.67^b^	61.78^b^	72.45^a^	75.27^ab^
SEM	3.192	1.538	1.749	1.452
*P-*value	< 0.001	< 0.001	0.007	0.011

SEM: standard error of the mean. Means with the same lowercase letters within a column are not significantly different (*P* < 0.05).

See footnotes at the bottom of Table 3.

#### Liver function

Measuring serum total proteins (TP) and albumin provides important insight into nutritional status and the functional integrity of organs such as the liver and kidneys. Reduced levels are often associated with malnutrition or hepatic and renal disorders, whereas elevated levels may occur in chronic inflammation or malignancy^56^. As shown in Table 5, serum TP levels were not significantly affected by either type of FM or probiotic supplementation. However, rats fed NDTFM exhibited the highest serum albumin levels, with a significant difference observed only compared with the NDLM group (*P* = 0.043). These findings partially align with previous reports suggesting that FM consumption may improve TP and albumin levels, likely due to protein hydrolysis by LAB into more digestible peptides and amino acids^57,58^. Other studies have shown that probiotic-enriched FM can significantly increase TP and albumin levels, particularly in female rats^59^. In the present study, BFM did not adversely affect TP or albumin compared with TFM, and supplementation with *B. bifidum* and *L. rhamnosus* did not confer additional benefits. ALAT and ASAT are also key biomarkers of liver function, released into circulation upon hepatocellular damage. As presented in Table 5, enzyme levels across all groups remained within normal ranges^60^. No significant differences in ALAT activity were observed among the NDBFM-, NDTFM-, and NDLM-fed rats. However, the NDPBFM group exhibited the lowest ALAT activity, showing a 14.23% reduction compared with the NDLM group (*P* = 0.002). Rats fed NDBFM exhibited the highest ASAT activity; however, this increase was significantly attenuated by probiotic supplementation (*P* < 0.001). This observation is consistent with the findings of Quan et al.^60^, who reported that a 22-day administration of MRPs, including furosine, pyralline, and 5-HMF, elevated ALAT, ASAT, ALP, and γ-glutamyltransferase activities in mice. The accumulation of these compounds may disrupt hepatic lipid and amino acid metabolism, contributing to liver and kidney dysfunction^61^. However, probiotics support liver health by enhancing gut barrier integrity, reducing inflammation, and promoting detoxification. They also improve lipid metabolism and antioxidant enzyme activity, thereby alleviating hepatic burden and oxidative stress^62^.

**Table 5:** Serum proteins, albumin levels, and liver enzymes of rats fed normal diets mixed with baked or traditional fermented milk, with or without probiotic bacteria, over 45 days.

Type of diet	Total protein (g/dL)	Albumin (g/dL)	ALAT (IU/L)	ASAT (IU/L)
NDLM	7.07^a^	3.72^b^	51.33^ab^	103.00^b^
NDTFM	7.41^a^	3.99^a^	55.00^a^	98.33^b^
NDPTFM	7.05^a^	3.87^ab^	48.83^b^	86.10^c^
NDBFM	7.44^a^	3.92^ab^	54.33^ab^	112.47^a^
NDPBFM	7.12^a^	3.79^ab^	44.00^c^	89.80^c^
SEM	0.233	0.036	1.082	2.047
*P-*value	0.391	0.043	0.002	< 0.001

SEM: standard error of the mean. Means with the same lowercase letters within a column are not significantly different (*P* < 0.05).

See footnotes at the bottom of Table 3.

#### Kidney functions

The effects of different FM types, with or without probiotic supplementation, on kidney function markers (urea and creatinine) are presented in Table 6. Except for the NDBFM group, all FM treatments showed a slight reduction in serum urea and creatinine levels compared with the NDLM group, consistent with findings by Alharbi et al.^63^, who reported improved renal markers in rats fed FM. These effects may be attributed to reduced oxidative stress and inflammation in kidney tissues^64^. Rats fed NDBFM exhibited a non-significant increase in serum urea compared with the NDLM group, but a significant elevation relative to the NDTFM, NDPTFM, and NDPBFM groups (*P* = 0.028). This elevation may be associated with high-temperature processing, which promotes Maillard reactions and whey protein denaturation. Although heat treatment can enhance protein digestibility, it may also accelerate the release and hepatic deamination of free amino acids, thereby increasing urea production^65^. MRPs can also increase the renal burden and contribute to oxidative stress. Elevated ASAT levels, particularly when accompanied by increased creatinine or urea, may indicate mild hepatic or renal stress associated with oxidized proteins or MRPs^66^. Conversely, Hillinger et al.^67^ suggest that elevated blood urea in the presence of normal creatinine levels is more likely attributable to increased protein catabolism or reduced hydration status than to direct renal impairment; however, the long-term physiological implications remain poorly characterized. Supplementation with *B. bifidum* or *L. rhamnosus* resulted in a significant reduction in serum urea levels in rats fed NDPBFM compared with the non-probiotic treatment. This effect may be attributed to the capacity of probiotics to attenuate oxidative stress and alleviate renal impairment associated with advanced glycation end products^64^.

**Table 6:** Kidney functions and oxidative stress biomarkers of rats fed normal diets mixed with baked or traditional fermented milk, with or without probiotic bacteria, over 45 days.

Type of diet	Kidney functions		Oxidative stress	
	Urea (mg/dL)	Creatinine (mg/dL)	MDA (nmol/ml)	NO (µmol/L)
NDLM	48.10^ab^	0.90^a^	8.19^a^	36.25^c^
NDTFM	45.23^b^	0.78^a^	7.48^ab^	43.35^b^
NDPTFM	46.00^b^	0.81^a^	6.39^bc^	33.73^c^
NDBFM	51.28^a^	0.73^a^	5.99^c^	52.62^a^
NDPBFM	45.06^b^	0.78^a^	5.39^c^	43.92^b^
SEM	0.789	0.045	0.252	2.346
*P-*value	0.028	0.124	0.002	< 0.001

SEM: standard error of the mean. Means with the same lowercase letters within a column are not significantly different (*P* < 0.05).

See footnotes at the bottom of Table 3.

#### Oxidative stress

As shown in Table 6, all FM groups exhibited a significant reduction in serum malondialdehyde (MDA) levels (*P* = 0.002), with the most pronounced decrease observed in the BFM groups, particularly NDPBFM. This effect may be attributed to the activity of *S. thermophilus* and *L. rhamnosus*, which are known to inhibit lipid peroxidation and scavenge free radicals, partly through the upregulation of antioxidant enzymes such as glutathione peroxidase and superoxide dismutase^68,69^. High-heat treatment of milk may also denature and degrade whey proteins and casein, leading to the release of peptides with antioxidant properties^70^. Lan et al.^71^ reported that short heat treatments may reduce the overall antioxidant capacity of milk. In contrast, intensive heat treatments that promote the formation of brown melanoidins through Maillard reactions can restore and even enhance antioxidant activity. Nitric oxide (NO) primarily promotes vasodilation, improving blood, oxygen, and nutrient delivery to vital organs^72^. Peptides from fermented milk have been reported to elevate serum NO in spontaneously hypertensive rats, contributing to antihypertensive effects^73^. Rats fed the NDBFM diet exhibited significantly higher serum NO levels than those in the NDLM group. Fermented dairy products have been reported to increase or restore bioavailable NO in the circulation, as bioactive peptides generated during fermentation can enhance vascular endothelial function and protect NO from oxidative degradation^74^. The NDBFM group showed significantly higher NO levels than both the NDLM and NDTFM groups (*P <* 0.001). This marked elevation in NO may be explained by two potential mechanisms. First, a synergistic effect between compounds generated during heat treatment (baking) and subsequent fermentation may enhance endothelial function and improve antioxidant status, thereby increasing NO bioavailability^75^. Second, NDBFM consumption may induce systemic inflammation, leading to overactivation of the inducible nitric oxide synthase (iNOS) pathway and excessive NO production during inflammatory responses^11^. However, serum NO levels decreased significantly following probiotic supplementation, declining from 43.35 to 52.62 µmol/L in the NDTFM and NDBFM groups to 33.73 and 43.92 µmol/L in the NDPTFM and NDPBFM groups, respectively. These findings suggest that the effect of probiotics on NO metabolism may depend on both the type of fermented milk and the probiotic strain used. Similar reductions in serum NO levels have been reported following the consumption of probiotic fermented milk products. Specifically, probiotic strains such as *Lb. paracasei* and *Lb. gasseri* have been shown to lower serum NO concentrations through the suppression of iNOS expression^76^. Hou et al.^77^ further reported that 5-HMF and certain MRPs, particularly melanoidins, may indirectly influence NO levels by reducing oxidative stress, thereby affecting NO synthesis and bioavailability.

## Conclusion

In conclusion, intensive heat treatment during BFM production (115 °C for 20 min) may reduce certain vitamins, particularly B-complex vitamins, and slightly decrease apparent viscosity. However, it also offers several advantages, including enhanced antioxidant activity, higher levels of flavor compounds such as diacetyl (which contributes to buttery flavor), improved textural smoothness, and the development of a characteristic brown color. The slight reduction in viscosity can be mitigated by incorporating probiotic strains that produce polysaccharides, thereby improving product texture. Biologically, BFM consumption exhibited several beneficial effects alongside mild metabolic alterations. BFM consumption, particularly when combined with probiotic supplementation, contributed to improved glucose regulation and reduced oxidative stress, as evidenced by lower blood glucose and MDA levels. However, BFM consumption may increase blood ASAT activity and urea levels. Probiotic supplementation appeared to exert more pronounced beneficial effects on these parameters and may have attenuated some of the mild metabolic shifts associated with BFM intake. Nevertheless, the present findings are limited in their ability to predict long-term health outcomes. Therefore, further studies are warranted to evaluate the long-term safety and physiological impact of heat-treated dairy products. Additional investigations incorporating molecular and histological analyses, as well as gut microbiota profiling, are essential to elucidate the underlying mechanisms and reinforce these findings.

## Data Availability

All data generated or analyzed during this study are included in this article.
